# Gray Transformation of Internal Medicine

**DOI:** 10.7759/cureus.9754

**Published:** 2020-08-15

**Authors:** Vishali Moond, Pratyush Shahi, Ashish Goel

**Affiliations:** 1 Internal Medicine, University College of Medical Sciences, Delhi, IND; 2 Orthopaedics, University College of Medical Sciences, Delhi, IND

**Keywords:** shift in admission trends, medicine ward, records analysis, geriatric care, geriatric medicine, health care system, health system reform

## Abstract

Aim

To study the profile of patients hospitalized in an internal medicine facility of a public hospital and explore if there is a shift in the age distribution of hospitalized patients toward an older age group.

Methods

The study was conducted at a tertiary care hospital in Delhi where the department of medicine has six units and a particular unit is in charge of the emergency room one day a week and every sixth Sunday. A total of 716 patients hospitalized in the medicine ward through the emergency services each Wednesday and every sixth Sunday (i.e, admission days of a particular unit) during a period for six months (November 2017 to April 2018) were retrospectively identified and their name, age, sex, comorbidities, final diagnosis, duration of hospital stay, and the outcome were noted. This was compared with similar data collected from a previous study conducted in the same setting in the years 2010-2011. The data were analyzed using Stata version 13 software (StataCorp, College Station, Texas). Findings were compared using the chi-squared test or t-test, wherever applicable.

Results

The mean age of patients hospitalized in 2010-2011 was 43.9 years, and this had increased to 48 years in 2018-2019 suggesting that the average patient being hospitalized in general medicine wards of a public hospital was now approximately five years older. Thirty-nine percent (39%) of the individuals admitted were more than 60 years of age in 2018-2019 as compared to 28.0% in 2010-2011. There was a significant rise in the cases of cardiovascular and gastrointestinal diseases and a decline in poisoning and infectious diseases from the year 2010-2011 to 2018-2019. A significantly higher prevalence of cardiovascular diseases, diabetes, and respiratory diseases was found in the current study among older adults.

Conclusion

There has been a significant shift in the distribution of hospitalized patients in the internal medicine in-patient wards toward the older age group during the last decade. Also, there is a significant difference between the disease profiles of older and young patients. A comprehensive approach to geriatric medicine should be introduced and followed.

## Introduction

People living longer all over the world proves the success of public health and infection control. With the overall improvement in the health care sector, life expectancy in India has increased from 62.3 years for males and 63.9 years for females in 2001-2005 to 67.3 years and 69.6 years, respectively, in 2011-2015 [1]. This increase in life expectancy, caused by immunization and control of communicable diseases, has been associated with an increase in non-communicable diseases due to long-standing comorbidities.

Older adults (aged more than 65 years) constitute 13% of the United States population and account for 43% of its annual inpatient care spending. This portion of the population is expected to continue using more health care services and increase from 13% to 19% of the population by 2030 [2]. Older adults are at greater post-hospitalization risk; one in 20 dies during hospitalization and another 20%-30% die within one year following hospital discharge [3]. The increase in life expectancy leads to the question of whether this population aging will be accompanied by sustained and improved health, improving quality of life, and sufficient social and economic resources. The answer lies partly in the ability of the families, communities, and health service delivery systems to provide optimal support to older persons.

Internal medicine services are significantly older than yesteryears and the spectrum of diseases is different than previously encountered. This fact, though largely felt, has yet not been established and requires a reorientation of training because internal medicine for the elderly is not the same as geriatric medicine. Leon Flicker stated the advantage of subspecialization as it allows the practitioner to focus on specific knowledge, skills, and attitudes that can achieve better patient outcomes [4]. Others such as Denaro CP and Mudge A didn’t agree with the idea of geriatric medicine [5].

The present study was carried out to assess the clinical and demographic profile of patients hospitalized to the internal medicine facility of a public hospital to find the age distribution of the patients hospitalized through the emergency services and compare this with similar previous data of a study conducting in the same setting in 2010-2011 [6].

## Materials and methods

The study was conducted at a tertiary care hospital in Delhi after being approved by the Institutional Ethics Board (approval number: IEC-HR/2018/36/84). The department of medicine at this hospital has six units and a particular unit is in charge of the emergency room one day a week and every sixth Sunday. This study retrospectively included 762 patients hospitalized to the medicine ward through emergency services each Wednesday and every sixth Sunday (i.e. admission days of a particular unit) during a period for six months (November 2018 to April 2019). The name, age, sex, comorbidities, final diagnosis, duration of hospital stay, and outcome of the participants were noted. The patients were excluded if there was uncertainty about the age or diagnosis. After excluding those with incomplete data, 716 patients were finally included for analysis. This was compared with similar data collected from a previous study conducted in the same setting in the year 2010-2011 during the same months.

The data were analyzed using Stata version 13 software (StataCorp, College Station, Texas). Findings were compared using the chi-squared test or t-test, wherever applicable.

## Results

A comparison of the patient profile between 2010 and 2011 & 2018 and 2019 is presented in Table 1. The proportion of patients more than 60 years had increased by 10.9% and the mean age of patients had increased by 4.1 years.

**Table 1:** Comparison of patient profile between 2010 and 2011 & 2018 and 2019

Patient profile	2010-2011	2018-2019
Age more than 60 years	28.2%	39.1%
Mean age of patients	43.9 years	48.0 years
Male sex	60.6%	56.2%

A comparison of the disease profile between 2010 and 2011 & 2018 and 2019 is presented in Table 2. There was a statistically significant increase in cases of cardiovascular and gastrointestinal diseases and a decrease in cases of poisoning and infections.

**Table 2:** Comparison of disease profile between 2010 and 2011 & 2018 and 2019 Data are presented as percentage * indicates a statistically significant difference (p<0.05)

Disease profile	2010-2011	2018-2019
Poisoning	6.7	2.8*
Infections	16.7	10.9*
Tuberculosis	6.8	7.1
Diabetes	7.2	7.9
Cardiovascular diseases	17.3	23.6*
Gastrointestinal diseases	6.2	9.6*
Autoimmune diseases	1.0	1.4
Hematological diseases	8.5	6.9
Neurological diseases	11.2	12.3
Nephrological diseases	8.1	6.5
Respiratory diseases	10.3	11.0

A comparison of the disease profile of older (more than 60 years of age) and younger patients in 2018-2019 is presented in Table 3. There was a statistically significant difference in cases of poisoning, infections, diabetes, and cardiovascular, gastrointestinal, hematological, and respiratory diseases between the two groups.

**Table 3:** Comparison of the disease profile of older (more than 60 years of age) and younger patients in 2018-2019 Data are presented as percentage * indicates a statistically significant difference (p<0.05)

Disease profile	Age < 60 years	Age > 60 years
Poisoning	4.4	1.1*
Infections	17.8	10.6*
Tuberculosis	8.6	6.3
Diabetes	7.8	12.9*
Cardiovascular diseases	12.5	25.6*
Gastrointestinal diseases	12.2	5.4*
Autoimmune diseases	2.4	1.4
Hematological diseases	9.0	3.7*
Neurological diseases	10.6	13.7
Nephrological diseases	5.2	4.6
Respiratory diseases	9.5	14.7*

## Discussion

This retrospective study evaluated the profile of patients hospitalized in the internal medicine facility of a public hospital in 2018-2019 and compared it with similar data collected in 2010-2011. The mean age of patients hospitalized was 43.9 years in 2010-2011 and had increased to 48 years in the present analysis, suggesting that the average patient being hospitalized in the general medicine ward of a public hospital was now approximately five years older. Thirty-nine percent (39%) of the individuals admitted in 2018-2019 were more than 60 years of age as compared to 28% in 2010-2011. This shows a significant increase in the number of elderly admissions in the ward in recent years.

The disease profile of hospitalized patients has also changed with time, showing a significant rise in the cases of cardiovascular and gastrointestinal diseases and a decline in poisoning and infectious diseases. Further, significantly higher prevalence of cardiovascular diseases, diabetes, and respiratory diseases among older adults may be due to the inadequate lifestyle adopted by the people of the elder age group, lack of physical activities in this technology advanced era, and polluted environment of the metropolitan cities. In our study, cardiovascular diseases comprised most elderly admissions, followed by respiratory and neurological diseases. 

Roberts et al. reported increasing rates of emergency department (ED) visits for elderly patients (aged 65-74 years) in the United States in the last decade of the previous century (1993-2003) and concluded that the visits for such patients increased 34% during the study period [7].

Chenore et al., in 2011, analyzed emergency hospitalizations to all the English hospitals and reported that older age (especially over 85 years) had been undervalued as a risk factor. Interestingly, half (49.6%) of all emergency hospitalizations in their study were older than 65 years and admissions rose progressively in each successive age band. Over the four years, the emergency admissions for the 65 to 84-year-old population rose by 16.3% and for the age 85-and-over population by 20.1% [8]. While our rate of increase was not as dramatic, the trends were quite similar.

Our study highlights the changing characteristics of the patients hospitalized in the medicine wards at a tertiary care hospital. The patients were included over a period of six months, and hence the seasonal variations in the disease presentation were taken into consideration. Also, the fact that participants were included on weekdays as well as weekends balanced out the variations in the patient profile and diseases. Our study highlights the role of age-related factors in healthcare-seeking behavior and emphasizes the increasing patient attendance at public hospitals, which calls for radical reforms and rational resource allocation. However, the study was limited to the medicine ward, that too to the admission days of a particular unit. It is recommended to assess if similar trends are being replicated in other departments and services provided by the hospital in a prospective longitudinal analysis.

## Conclusions

In a retrospective study of patients hospitalized in the medicine wards through the emergency department, we noted that there has been a significant shift in the distribution of hospitalized patients in the internal medicine in-patient wards toward the older age group during the last decade. Also, there is a significant difference between the disease profiles of older and young patients. The changing age distribution and disease profile indicate the current trends and highlight the future of medical practice. Approaching the management of older patients as merely ‘internal medicine for the elderly’ should no longer be followed and a comprehensive approach to geriatric medicine should be introduced and followed.
